# The Negative Regulative Roles of *BdPGRPs* in the Imd Signaling Pathway of *Bactrocera dorsalis*

**DOI:** 10.3390/cells11010152

**Published:** 2022-01-04

**Authors:** Ping Zhang, Zhichao Yao, Shuai Bai, Hongyu Zhang

**Affiliations:** State Key Laboratory of Agricultural Microbiology, Institute of Urban and Horticultural Entomology, College of Plant Science and Technology, Huazhong Agricultural University, Wuhan 430070, China; 15927410478@163.com (P.Z.); yzc19880118@sina.com (Z.Y.); 15764278717@163.com (S.B.)

**Keywords:** Imd pathway, PGRPs, bacterial infection, negative regulators, gene identification

## Abstract

Peptidoglycan recognition proteins (PGRPs) are key regulators in insects’ immune response, functioning as sensors to detect invading pathogens and as scavengers of peptidoglycan (PGN) to reduce immune overreaction. However, the exact function of PGRPs in *Bactrocera dorsalis* is still unclear. In this study, we identified and functionally characterized the genes *BdPGRP-LB*, *BdPGRP-SB*_1_ and *BdPGRP-SC*_2_ in *B. dorsalis*. The results showed that *BdPGRP-LB*, *BdPGRP-SB*_1_ and *BdPGRP-SC*_2_ all have an amidase-2 domain, which has been shown to have *N*-Acetylmuramoyl-l-Alanine amidase activity. The transcriptional levels of *BdPGRP-LB* and *BdPGRP-SC*_2_ were both high in adult stages and midgut tissues; *BdPGRP-SB*_1_ was found most abundantly expressed in the 2nd instar larvae stage and adult fat body. The expression of *BdPGRP-LB* and *BdPGRP-SB*_1_ and *AMPs* were significantly up-regulated after injury infected with *Escherichia coli* at different time points; however, the expression of *BdPGRP-SC*_2_ was reduced at 9 h, 24 h and 48 h following inoculation with *E. coli*. By injection of dsRNA, *BdPGRP-LB*, *BdPGRP-SB*_1_ and *BdPGRP-SC*_2_ were knocked down by RNA-interference. Silencing of *BdPGRP-LB*, *BdPGRP-SB*_1_ and *BdPGRP-SC*_2_ separately in flies resulted in over-activation of the Imd signaling pathway after bacterial challenge. The survival rate of the *ds-PGRPs* group was significantly reduced compared with the *ds-egfp* group after bacterial infection. Taken together, our results demonstrated that three catalytic *PGRPs* family genes, *BdPGRP-LB*, *BdPGRP-SB*_1_ and *BdPGRP-SC*_2_, are important negative regulators of the Imd pathway in *B. dorsalis*.

## 1. Introduction

Insects come into contact with many kinds of pathogenic microorganisms from their habitat, and therefore insects have involved a strong innate immune system to resist microbial challenge. This system immediately responds against invading pathogens, and consists of cellular and humoral immune responses [1]. The activation of a series of antimicrobial defense mechanisms relies on a microbial sensing system of pattern-recognition receptors (PRRs) [2]. In insects, peptidoglycan recognition proteins (PGRPs) are a major class of PRRs that can recognize peptidoglycan (PGN), the specific component of the cell wall in both Gram-positive and Gram-negative bacteria [3,4]. PGN is a polymer with alternating N-acetylglucosamine and N-acetylmuramic acid residues that are cross-linked to each other by short peptide bridges; Gram-negative bacteria and Gram-positive *Bacilli* have DAP type PGN, unlike Gram-positive bacteria, which have Lys type PGN [5,6]. PGRP was first discovered in silkworms (*Bombyx mori*) in the late 1990s. PGRP was confirmed to have the ability to trigger a series of prophenoloxidase cascades after binding to different types of peptidoglycans [7]. With the progress of genome projects for different species, PGRP and its homologues have been identified in animals ranging from insects to mammals [8,9,10,11]. PGRPs are highly conserved from insects to mammals, which share a conserved 160 amino acid domain with similarities to the bacteriophage T7 lysozyme, a zinc-dependent amidase that hydrolyzes peptidoglycan [4].

Studies of PGRPs have focused on *D. melanogaster. Drosophila* has 13 PGRP genes which can encode 20 PGRP proteins; PGRPs can be divided into catalytic PGRPs and non-catalytic PGRPs according to their function [8]. Noncatalytic PGRPs (PGRP-SA, SD, LA, LC, LD, LE and LF) can only bind to peptidoglycan and lack amidase activity due to the absence of key cysteine residues for zinc binding, which are crucial for sensing of bacteria and activating immune pathways in the immune system. By contrast, catalytic PGRPs (PGRP-SC1a/b, SC_2_, LB and SB_1/2_) hydrolyze peptidoglycan by cleaving the amide bond between MurNAc and the peptidic bridge, leading to a termination of immune response [12,13]. The amidase PGRPs function as key immunoregulatory factors, regulating the immune response by cleaving peptidoglycan and existing directly as a bactericide [14]. In *D. melanogaster*, amidase PGRPs reduce the expression level of AMPs by degrading peptidoglycan and downregulating the immune response [13]. *PGRP-LB* deletion mutant and *Pirk* deletion mutant, and to a lesser extent *PGRP-SC* single deletion mutant flies showed reductions in mean lifespan compared to wild-type after *Ecc15* (*Erwinia carotovora carotovora 15*) infection. The excessive death of null mutants was due to their own excessive immune response rather than the accumulation of conditional pathogens, which has been further confirmed in [13]. *DmPGRP-LB* with amidase can downregulate the immune response by converting the Gram negative PGN to non-immunostimulatory fragments [6]. *DmPGRP-SC*_2_ was inhibited by FOXO with age, leading to immune system disorders and intestinal microbial disorders [15]. *DmPGRP-SB*_1_ has an amidase activity against DAP-type PGN, while *DmPGRP-SB*_1_ and *SB*_2_ are, at most, only marginally involved in the regulation of the Imd pathway [13,16].

The oriental fruit fly *Bactrocera dorsalis* (Hendel) is a destructive polyphagous and invasive insect pest of tropical and subtropical fruits and vegetables [17]. Owing to its vast adaptability, high reproduction potential and invasive capacity, *B. dorsalis* has been one of the world’s most invasive and polyphagous pests of agriculture [18]. *B.* *dorsalis* larvae live in rotten fruits and are more likely to be exposed to pathogenic bacteria. Indeed, *B.* *dorsalis* is emerging as a good material for research into immunity [19,20] and the role of immunity in microbiota homeostasis [21]. Although the functions of *PGRPs* have been shown in a number of insects, especially in *D. melanogaster* [16,22,23,24,25] and in other insects such as *Musca domestica*, *Sitophilus zeamais, Rhynchophorus ferrugineus* [26,27,28] as well, there is no clear picture of the role of *PGRPs* in *B. dorsalis.*

In this study, we cloned *BdPGRP-LB*, one isoform of *PGRP-SB* (*BdPGRP-SB*_1_), and one isoform of *PGRP-SC* (*BdPGRP-SC*_2_). The expression profiles of the *BdPGRP-LB*, *BdPGRP-SB*_1_ and *BdPGRP-SC*_2_ genes in different developmental stages and adult tissues were examined by real-time quantitative polymerase chain reaction (qRT-PCR). We monitored the immune response of *BdPGRPs* after adults were infected with the Gram-negative bacteria *E.* *coli*, and revealed the important negative roles of the *BdPGRP-LB, BdPGRP-SB*_1_ and *BdPGRP-SC*_2_ genes in the Imd pathway of *B. dorsalis* using RNA interference methods.

## 2. Materials and Methods

### 2.1. Experimental Insects

*B. dorsalis* was collected from Guangzhou, China and reared more than 20 generations at the Institute of Urban and Horticultural Pests at Huazhong Agricultural University, Wuhan, as described by Li et al. [17]. The newly emerged adults were reared in cages under the following conditions: 28 ± 1 °C, 70–80% relative humidity, 12 h/12 h light/dark cycle; adults’ artificial diet contained 2.5% yeast extract, 7.5% sugar, 2.5% honey, 0.5% agar and 87% water; eggs and larvae were fed on bananas. 

### 2.2. Cloning and Analysis of the BdPGRP Genes

Total RNA was extracted from *B. dorsalis* with RNAiso^TM^ Plus reagent (TaKaRa, Otsu, Shiga, Japan) following the manufacturer’s instructions. Ten newly emerged adults of *B. dorsalis* with a sex ration at 1:1 were homogenized in 1mL RNAiso with a burnisher (Shanghai Jingxin Industrial Development Co., Ltd., Shanghai, China) at 70 Hz/s for 60 s at 10 s intervals. The purity of the RNA was analyzed using a NanoDrop 1000 Spectrophotometer (Thermo Fisher Scientific, Waltham, MA, USA) and the quality of RNA was tested by 1.0% agarose gel electrophoresis at voltage 120 V, 20 min in TAE buffer. First strand cDNA was synthesized from 1 μg RNA using the PrimeScript™ RT reagent Kit with gDNA Eraser Kit (TaKaRa). Then, the cDNA was served as template. The amplification of 3′- and 5′- cDNA ends of *BdPGRP-SC*_2_ was conducted with the 3′-Full RACE Core Set (Cat. # 6121) (TaKaRa, Otsu, Shiga, Japan) and 5′-Full RACE Kit (Cat. # 6122) (TaKaRa, Otsu, Shiga, Japan) according to the manufacturer’s instructions. Primers for RACE were designed according to the fragment sequence from transcriptome of *B. dorsalis*. The sequence of *BdPGRP-LB* and *BdPGRP-SB*_1_ were obtained from the NCBI database (Genebank: GAKP01019367; GAKP01007643). PCR conditions were 94 °C 3 min; 94 °C 30 s, 55 °C 30 s, 72 °C 60 s for 35 cycles; 72 °C 10 min. PCR was carried out in a volume of 25  μL consisting of 12.5 μL PCR Mix (Biomed, Beijing, China), 100  nM of each primer and 1 μg of cDNA. PCR products were purified with AxyPrep DNA Gel Extraction Kit (AXYGEN, Union City, CA, USA) and then cloned into pEASY-T1 Cloning Vector (TransGen, Beijing, China) and sequenced.

The nucleotide and protein sequences were analyzed with DNAMAN 6.0 (Lynnon Corporation, Quebec, QC, Canada). Nucleotide sequence alignment used the blast online tools (https://blast.ncbi.nlm.nih.gov/Blast.cgi (accessed on 18 January 2021)). Amino acid sequence alignment was analysed using DNAMAN software. The functional protein predictions were analyzed using online tools (http://smart.embl-heidelberg.de/smart/set_mode.cgi (accessed on 18 January 2021)). A phylogenic neighbour-joining (NJ) tree was constructed with the Mega7 software package (Mega, Auckland, New Zealand). The sequence data were transformed into a distance matrix. One thousand bootstraps were performed for the NJ tree to check the repeatability of the results.

### 2.3. Development Stage and Tissue Expression Profiles

The expression profile was analysed by qRT-PCR. Different development stages of *B.* *dorsalis* were collected: eggs, first instar larvae, second instar larvae, third instar larvae, early pupae (48 h after pupation), old pupae (48 h before eclosion), adults (sex ration at 1:1) before mating (2–3 days after eclosion), and adults (sex ration at 1:1) after mating (13–15 days after eclosion). For eggs, five independent cohorts of every 50 eggs were collected as biological replicates. For larvae, pupae, and adults, five independent cohorts of every ten individuals were collected as biological replicates. For different tissue collection, the adults (2–3 days after eclosion) were sterilized for 2–5 min in 75% alcohol, washed in DEPC-water three times and then dissected in phosphate buffer saline (137 mM NaCl, 2.7 mM KCl, 10 mM Na_2_HPO_4_, 2 mM KH_2_PO_4_, pH 7.4). The different tissues examined included the head, midgut, hindgut, Malpighian tubule, fat body, ovaries and testes. Five independent cohorts of every 30 flies were dissected and used as biological replicates. All samples were homogenized in 1 mL RNAiso^TM^ Plus (TaKaRa, Otsu, Shiga, Japan) as described above, followed by RNA extraction and cDNA synthesis. 

### 2.4. Bacterial Preparation and Infection Bioassays

*Escherichia coli* DH5α used in this experiment were stored in the Institute of Urban and Horticultural Entomology, Huazhong Agricultural University. A Gram-negative bacterium, *E. coli* has DAP type PGN, and the Imd pathway can be activated by DAP type PGN [29]. *E. coli* were cultivated in 400mL LB (Luria–Bertani) medium at 37 °C with shaking 220 r/min for 3–5 h until the concentration of OD 600 = 1 (~5 × 10^8^ colony-forming units (CFUs)), as previous described [21]. Then, the bacteria cultures were centrifuged at 3600× *g* for 5 min at room temperature and washed two times with phosphate buffer saline. For systemic infection, the bacteria pellets were resuspended in LB and adjusted to a certain concentration (OD600 = 400) for infection.

For infection bioassays, 250 newly emerged flies (within three days following eclosion) were collected in boxes. The glass needles which were prepared with a puller at heat level 60.8 (PC-10, Narishige, Tokyo, Japan) were used to dip into the bacteria pellet (OD600 = 400) or LB medium (the Control) for 30 s, and then the thorax of ice anaesthetized adult flies was inoculated and ten whole body samples were collected at 1 h, 3 h, 6 h, 9 h, 12 h, 24 h, 48 h after infection with a sex ratio of 1:1. The experiment was repeated three times.

### 2.5. Double Strain RNA Synthesis and RNAi

PCR amplification was carried out with primers of gene fragments containing T7 polymerase promoter (GGATCCTAATACGACTCACTATAGG). The *egfp* fragment which was used as a control was also amplified from Pub. nls. EGFP (Provided by Dr. Handler, USDA). The primers used to amplify the specific DNA fragments are listed in Table 1. PCR products were purified with an AxyPrep DNA Gel Extraction Kit (AXYGEN, USA) and then used as the template for double-stranded RNA synthesis by using a T7 RiboMAX™ Express RNAi System (Promega, Madison, WI, USA) as per the manufacturer’s instructions. The dsRNA pellet was resuspended in RNase-free water and quantified at 260 nm using a Nanodrop 1000 spectrophotometer (Thermo Fisher Scientific, Waltham, MA, USA). The quality of dsRNA was tested by 1.2% agarose gel electrophoresis at voltage 120 V, 20 min in TAE buffer.

Microinjection was performed using an Eppendorf micromanipulation system (Microinjector for cell biology, FemtoJet 5247, Hamburg, Germany). The injection condition was set to a Pi of 300  hpa and a Ti of 0.3 s. The needles for microinjection were made with a puller at heater level 60.8 (PC-10, Narishige, Tokyo, Japan) as previous described [21]. Each fly (three days after eclosion) was injected with 1 μL dsRNA at a concentration of 2000 ng/μL for the gene knockdown experiment. After injection, adult flies were transferred to a 17 cm × 8 cm × 7 cm plastic box and fed an artificial diet.

### 2.6. Investigation of the RNAi Off-Target Effect and RNAi Efficiency

Based on sequence similarity, numerous off-targets are predicted to occur in RNAi experiments [30]. *BdPGRP-LB*, *BdPGRP-SB*_1_ and *BdPGRP-SC*_2_ all belong to a PGRP family with high sequence homology. It is critical to investigate the RNAi off-target effect during *PGRP* gene RNAi experiments. To ensure the other *PGRPs* transcripts were not affected by one *PGRP* gene RNAi, the mRNA expression level was examined by qRT-PCR. qRT-PCR was performed with iQTM SYBR^®^ Green Supermix (Bio-Rad, Berkeley, CA, USA) on Bio-Rad iQ5 (Bio-Rad, Berkeley, CA, USA). The 20 μL reactions contained 10 μL 2 × Master Mix, 2 μL cDNA (diluted 1:10), 0.8 μL 10 pmol forward and reverse primers and 6.4 μL double-distilled water. The PCR program was preincubated at 95 °C for 3 min followed by 40 cycles of denaturation at 95 °C for 10 s and annealing at 60 °C for 30 s. When *BdPGRP-LB* was knocked down at 24 h after RNAi, the expression of *BdPGRP-SB*_1_ and *BdPGRP-SC*_2_ was detected at the same time; when *BdPGRP-SB*_1_ was knocked down at 24 h after RNAi, the expression of *BdPGRP-LB* and *BdPGRP-SC*_2_ was detected at the same time; and when *BdPGRP-SC*_2_ was knocked down at 24 h after RNAi, the expression of *BdPGRP-LB* and *BdPGRP-SB*_1_ was detected at the same time.

### 2.7. The Effects of Knockdown of BdPGRPs on the Imd Pathway Response to Bacterial Challenge 

To explore the effects of silencing *PGRPs* in the Imd pathway of *B. dorsalis*, *E. coli* were inoculated at 24 h after RNAi. There were three experimental groups: the control group was inoculated with LB medium 24 h after injection with *ds-egfp*; the *ds-egfp* group was infected with *E. coli* (OD600 = 400) after injection with *ds-egfp;* and the *ds-PGRPs* group was infected with *E. coli* (OD600 = 400) after injection with *ds-PGRPs*. Then, the expression of *Dpt,* a marker of Imd pathway activation, was detected at 6 h, 12 h, 24 h and 48 h after infection.

### 2.8. Survival Assay of B. dorsalis

24 h after dsRNA injection (separately or combined 3 *BdPGRPs* genes), insects that were alive in the control and treatment groups were individually challenged with *E.* *coli* by inoculation with bacteria resuspended in LB (Luria–Bertani) (refer to bacterial infection bioassays). Infected flies were placed into new boxes and these boxes into thermostatic incubator at 28 ± 1 °C, 70–80% relative humidity, 12 h/12 h light/dark cycle, and fed with artificial diet. The mortality of *B. dorsalis* adults was monitored daily and dead insects were recorded and removed from the boxes.

### 2.9. Quantitative Real-Time PCR

All tested samples of RNA were extracted with RNAiso^TM^ Plus (TaKaRa) following the manufacturer’s instructions; refer to Section 2.2. The purity of the RNA was analyzed using a NanoDrop 1000 Spectrophotometer (Thermo Fisher Scientific Inc., USA) and the quality of RNA was tested by 1.0% agarose gel electrophoresis at voltage 120 V, 20 min in TAE buffer. cDNA was synthesized using the PrimeScript™ RT reagent Kit with gDNA Eraser (TaKaRa). The first-strand complementary DNA (cDNA) of each pool was synthesized from 1 μg of total RNA using a two-step cDNA synthesis kit (Takara) with the gDNA eraser to remove residual DNA contamination. qRT-PCR was performed with iQTM SYBR^®^ Green Supermix (Bio-Rad, USA) on Bio-Rad iQ5 (Bio-Rad, USA). The 20 μL reactions contained 10 μL 2 × Master Mix, 2 μL cDNA (diluted 1:10), 0.8 μL 10 pmol forward and reverse primers and 6.4 μL double-distilled water. The PCR program was preincubated at 95 °C for 3 min, followed by 40 cycles of denaturation at 95 °C for 10 s and annealing at 60 °C for 30 s. Melting curve analysis was performed at the end of the program to confirm the specificity of the primers. *BdRpl32* was chosen as the reference gene. To determine the amplification efficiencies, a standard curve was established for each primer pair with serial dilutions of cDNA (1/1, 1/10,1/100,1/1000,1/10^4^, 1/10^5^). Every sample had three technical replicates. The relative gene expression data were analyzed using a 2^−ΔΔCT^ method and the data were normalized to the reference gene *Rpl32* for mRNA expression analysis [31]. The qPCR primers are listed in Table 1.

### 2.10. Statistical Analyses

Comparisons between the means of two independent groups were performed with Student’s t-test, and multiple comparisons of results from experimental replicates were analyzed by one-way analysis of variance (ANOVA) and Turkey’s test using SPSS 16.0 (IBM Corporation, Somers, NY, USA). Survival statistical analysis was based on Log-rank (Mantel–Cox) test. The plots were handled with Excel (Microsoft, Redmond, WA, USA) and GraphPad Prism 7 (GraphPad Software Inc., San Diego, CA, USA).

## 3. Results

### 3.1. Sequence Features, Phylogenetic Tree and Functional Domain Prediction of PGRPs in B. dorsalis

**Figure 1:**
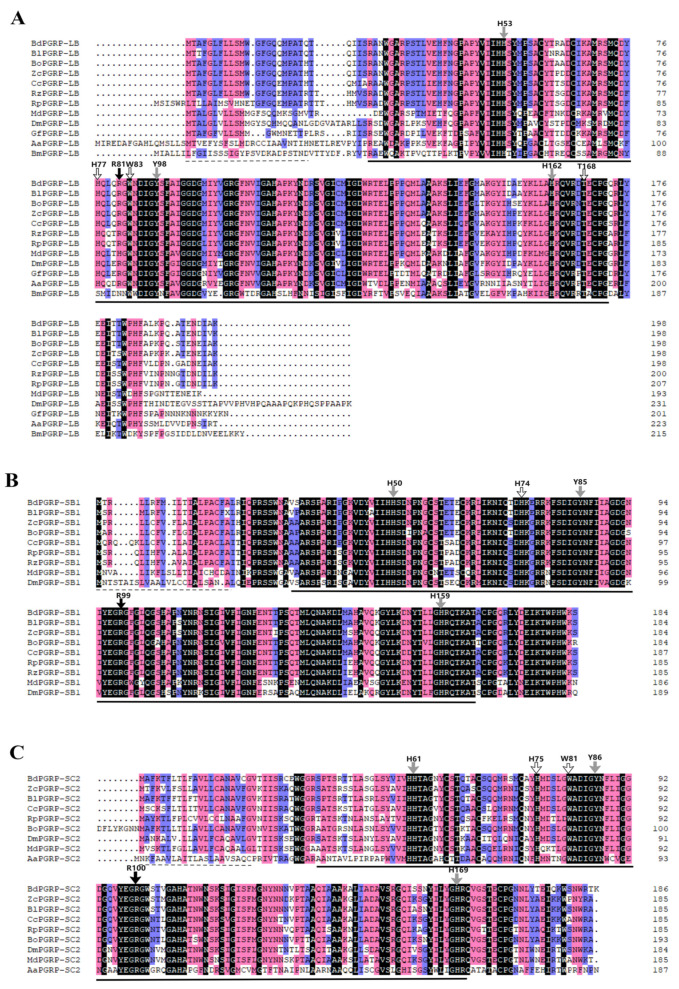
Amino acid sequence alignment of *BdPGRPs* with that of homologous genes in other insect species. (**A**) Multiple alignments of *PGRP-LB*. *BdPGRP-LB* was aligned with *Bactrocera latifrons PGRP-LB* (XP_018789449.1), *Bactrocera oleae PGRP-LB* (XP_014091181.2), *Zeugodacus cucurbitae PGRP-LB* (XP_011197144.1), *Ceratitis capitate PGRP-LB* (XP_004518089.1), *Rhagoletis zephyria PGRP-LB* (XP_017470705.1), *Rhagoletis pomonella PGRP-LB* (XP_036322481.1), *Aedes aegypti PGRP-LB* (XP_021709443.1), *Drosophila melanogaster PGRP-LB* (NP_731575.1), *Bombyx mori PGRP-LB* (XP_012548100.1), *Musca domestica PGRP-LB* (XP_005180889.1), and *Glossina fuscipes PGRP-LB* (ACI22620.1). (**B**) Multiple alignments of *PGRP-SB*. *BdPGRP-SB*_1_ was aligned with *B. latifrons PGRP-SB* (XP_018789286.1), *B. oleae PGRP-SB* (XP_014099773.1), *Z. cucurbitae PGRP-SB* (XP_011181375.1), *C. capitate PGRP-SB* (XP_004537949.1), *R. zephyria PGRP-SB* (XP_017486043.1), *R. pomonella PGRP-SB* (XP_036336342.1), *D. melanogaster PGRP-SB* (CAD89135.1), *M. domestica PGRP-SB* (NP_001295929.1), and *B. mori PGRP-SB* (XP_004929843.1). (**C**) Multiple alignments of *PGRP-SC*_2_. *BdPGRP-SC*_2_ was aligned with *B. latifrons PGRP-SC*_2_ (XP_018798904.1), *B. oleae PGRP-SC*_2_ (XP_014085196.2), *C. capitate PGRP-SC*_2_ (XP_004520319.1), *Z. cucurbitae PGRP-SC*_2_ (XP_011180165.1), *R. pomonella PGRP-SC*_2_ (XP_036334551.1), *M. domestica PGRP-SC*_2_ (XP_005184140.3), *D. melanogaster PGRP-SC*_2_ (CAD89184.1), *A. aegypti PGRP-SC*_2_ (XP_011492940.1), and *B. mori PGRP-SC*_2_ (XP_004929814.1). The identical amino acids are shown against a black background; 75% conserved amino acids are shown against a pink background; 50% conserved amino acids are shown against a blue background. The signal peptides are indicated by dashed lines. The amidase domains are indicated by solid lines. Black arrows indicate the amino acid residues required for the recognition of DAP-type peptidoglycan. Grey arrows indicate the amino acid residues required for Zn^2+^ binding. White arrows indicate the amino acid residues required for amidase activity.

A 564 bp nucleotide fragment of *BdPGRP-SC*_2_ was obtained by RACE; the GenBank accession number of the fragment of *BdPGRP-SC*_2_ is MW538960. The gene encoded a 188-amino acid protein. Both amino acid sequence alignment (Figure 1) and protein prediction results (Supplementary Figure S1) indicated that *BdPGRP-LB*, *BdPGRP-SB*_1_ and *BdPGRP-SC*_2_ all have a type 2 amidase domain, which has been shown to have N-acetylmuramoyl-L-alanine amidase activity. Amino acid sequence analysis showed that *BdPGRP-LB*, *BdPGRP-SB*_1_ and *BdPGRP-SC*_2_ all have conserved amino acid Arg, which is necessary for the recognition of DAP-type peptidoglycan [32]. *BdPGRP-LB* has three conserved histidines, H53, H77, H162, one conserved tryptophan, W83, one conserved tyrosine, Y98 and one conserved threonine, T168, which are required for Zn^2+^ binding and amidase activity (Figure 1A). *BdPGRP-SB*_1_ has conserved H50, H74, H159 and Y85 for amidase activity (Figure 1B). *BdPGRP-SC*_2_ has conserved H61, H75, H169, W81 and Y86 for Zn^2+^ binding and amidase activity (Figure 1C). These results indicate that *BdPGRP-LB*, *BdPGRP-SB*_1_ and *BdPGRP-SC*_2_ belong to the catalytic PGRPs. A phylogenetic tree was constructed to determine the evolutionary relationships with *PGRPs* from several other insect species of Diptera (Supplementary Figure S2). The results show that *BdPGRP-LB*, *BdPGRP-SB*_1_ and *BdPGRP-SC*_2_ from different species converge in a clade; respectively, this indicates that *PGRP-LB*, *PGRP-SB*_1_ and *PGRP-SC*_2_ evolved independently. In addition, all three *BdPGRPs* of *B*. *dorsalis* were closest to those of *B. latifrons* in evolution of the three genes (Supplementary Figure S2).

### 3.2. The Expression Profilings of BdPGRPs in B. dorsalis

**Figure 2:**
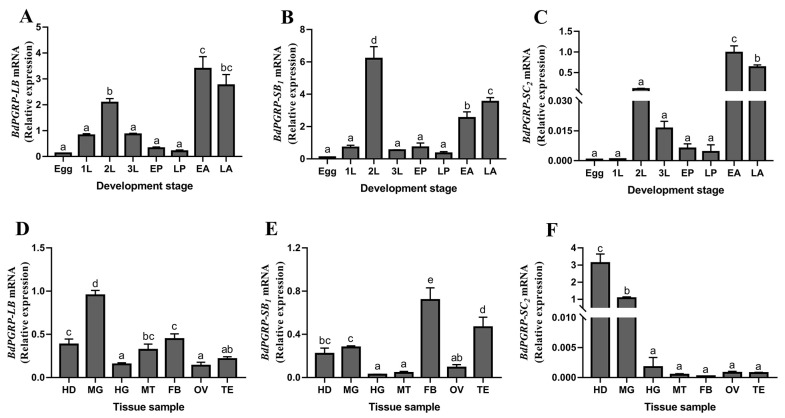
Expression profiles of *BdPGRPs* in *B.dorsalis*. (**A**) Relative expression of *BdPGRP-LB* at different development stages. (**B**) Relative expression of *BdPGRP-SB*_1_ at different development stages. (**C**) Relative expression of *BdPGRP-SC*_2_ at different development stages. (**D**) Relative expression of *BdPGRP-LB* from different tissue samples. (**E**) Relative expression of *BdPGRP-SB*_1_ from different tissue samples. (**F**) Relative expression of *BdPGRP-SC*_2_ from different tissue samples. *B.* *dorsalis* was collected at various developmental stages: 1 L, 1st instar larvae; 2 L, 2nd instar larvae; 3 L, 3rd instar larvae; EP, early pupal stage; LP, late pupal stage; EA, newly emergence adults; LA, late adult stage. Different adult tissues were collected: HD, head; MG, midgut; HG, hindgut; MT, Malpighian tube; FB, fatbody; OV, ovary; TE, testis. Multiple comparisons were carried out with one-way analysis of variance (ANOVA) and Turkey’s test in SPSS 16.0. Different lower-case letters indicate a significant difference at the level of *p* < 0.05 and a confidence interval of 95%. The relative gene expression data were analyzed using a 2^−ΔΔCT^ method and the data were normalized to reference gene *Rpl32*.

qPCR was performed to detect the expression pattern of *BdPGRP-LB*, *BdPGRP-SB*_1_ and *BdPGRP-SC*_2_ in different development stages and in various tissues using the primers listed in Table 1. *BdPGRPs* can be detected across the life stage of *B. dorsalis*, and the expression levels of *BdPGRP-LB*, *BdPGRP-SB*_1_ and *BdPGRP-SC*_2_ were all highly expressed in the adult stage and in the second instar larvae stage (Figure 2A–C), and expressed weakly in the egg, 1st instar larvae, 3rd instar larvae and pupa stages (Figure 2A–C).

Tissue profiles of *BdPGRP-SB*_1_*, BdPGRP-LB* and *BdPGRP-SC*_2_ were also analyzed by qRT-PCR. In contrast to the weak expression observed in the hindgut and ovary, the *BdPGRP-LB* and *BdPGRP-SB*_1_ were primarily distributed in the head, midgut and fatbody (Figure 2D,E). *BdPGRP-LB* was also highly expressed in the Malpighian tubules (Figure 2D), which are vital immune response-related sites [33]. The high expression of *BdPGRP-SB*_1_ observed in the testis suggested they may have an important role in reproductive development of *B. dorsalis* (Figure 2E). Interestingly, the tissue specific expression indicated that *BdPGRP-SC*_2_ had higher expression levels in the head and midgut than in other tissues (Figure 2F). The varied expression of *BdPGRP-LB*, *BdPGRP-SB*_1_ and *BdPGRP-SC*_2_ in different developmental stage and tissues suggests that *BdPGRPs* may play distinct roles in *B. dorsalis*.

### 3.3. Responses of BdPGRPs to Systemic Bacterial Infection

**Figure 3:**
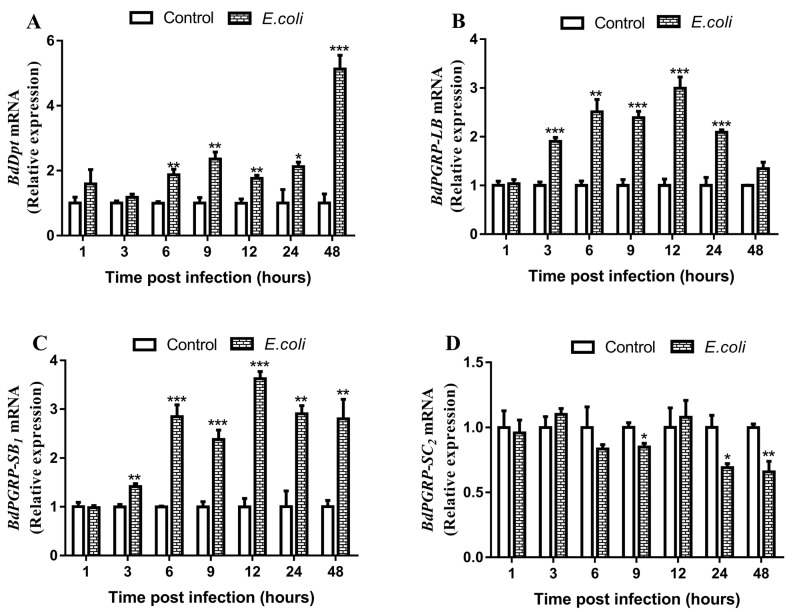
Responses of *Dpt* and *BdPGRPs* to opportunistic pathogen *E. coli* challenges. Relative expression of *Dpt* (**A**), *BdPGRP-LB* (**B**), *BdPGRP-SB*_1_ (**C**), and *BdPGRP-SC*_2_ (**D**) after infection with *E. coli* at different time points, respectively. The data are expressed as mean ± SEM and the mean refers to the average of four biological replicates for each sample. Statistical analysis was based on Student’s *t*-test. * *p* < 0.05; ** *p* < 0.01; *** *p* < 0.001. The relative gene expression data were analyzed using a 2^−ΔΔCT^ method and the data were normalized to reference gene *Rpl32*.

To investigate the *BdPGRPs* response to bacterial challenge, the expression of *BdPGRP-LB*, *BdPGRP-SB*_1_ and *BdPGRP-SC*_2_ at 1 h, 3 h, 6 h, 9 h, 12 h, 24 h and 48 h after *E. coli* thorax inoculation was monitored in whole insects. The results showed that there was a significant increase of the expression of *BdPGRP-LB* and *BdPGRP-SB*_1_ during 3–24h infection, with a 1.90–2.99-fold and 1.40–3.62-fold increase, respectively (Figure 3B,C). Unexpectedly, there was a decrease of *BdPGRP-SC*_2_ at 9 h, 24 h and 48 h following inoculated with *E. coli*, and no response at other times post infection (Figure 3D). We also found an immune response of effector genes of the Imd pathway to *E. coli* infection; there was a 1.76–5.13-fold increase of the expression of the antimicrobial peptide gene *Diptericin* at 6h–48h post infection (Figure 3A). The relative expression of other antimicrobial peptide genes including *AttacinA*, *AttacinB*, *AttacinC* and *Cecropin* were also induced by *E. coli* infection, thus confirming the strong immunogenic nature of *E. coli* infection in *B. dorsalis*. (Supplementary Figure S3A–D). The above results indicate that inoculation with *E. coli* can immediately activate the immune response of the Imd pathway in *B. dorsalis*.

### 3.4. RNA Interference (RNAi) of BdPGRPs

**Figure 4:**
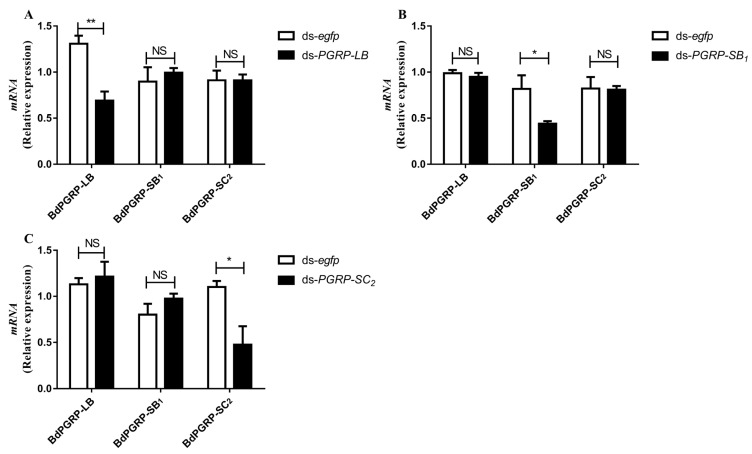
Off-target detection after dsRNA injection. (**A**) Influence of silencing *BdPGRP-LB* on expression of *BdPGRP-SB*_1_ and *BdPGRP-SC*_2_. (**B**) Influence of silencing *BdPGRP-* *SB*_1_ on expression of *BdPGRP-LB* and *BdPGRP*-SC_2_. (**C**) Influence of silencing *BdPGRP-* *SC*_2_ on expression of *BdPGRP-LB* and *BdPGRP-SB*_1_. All error bars represent the SEM of the mean of three independent biological replicates. Statistical analysis was based on Student’s *t*-test. * *p* < 0.05; ** *p* < 0.01; NS, no significant difference; *p* > 0.05. The relative gene expression data were analyzed using a 2^−ΔΔCT^ method and the data were normalized to reference gene *Rpl32*.

Based on sequence similarity (Figure 1), numerous off-targets are predicted to occur in RNAi experiments [30]. To test whether potential off-target effects of *ds-PGRPs* exist, the off-target effect was analysed by qRT-PCR (Figure 4). When *BdPGRP-LB* RNAi was performed, the relative expression of *BdPGRP-SB*_1_ and *BdPGRP-SC*_2_ was also detected one day after dsRNA injection. Results showed that the expression of *BdPGRP-SB*_1_ and *BdPGRP-SC*_2_ were not affected by *BdPGRP-LB* knock down (Figure 4A). *BdPGRP-SB*_1_ RNAi did not affect the relative expression of *BdPGRP-LB* and *BdPGRP-SC*_2_ (Figure 4B), nor did *BdPGRP-SC*_2_ RNAi (Figure 4C). These results suggested that there was no off-target effect in the *BdPGRPs* knockdown experiment in *B. dorsalis*.

**Figure 5:**
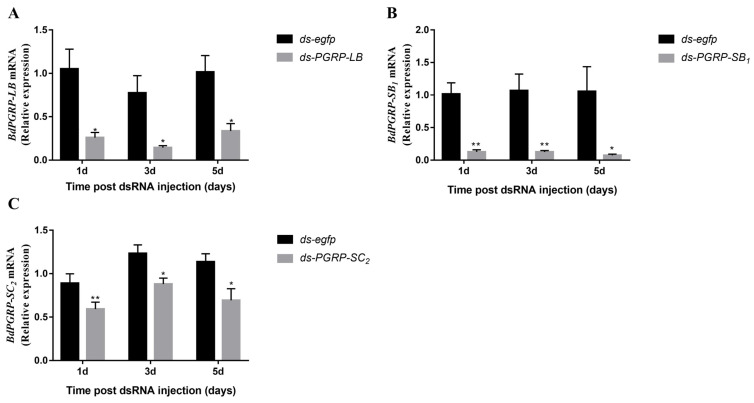
RNA interference efficiency of *BdPGRPs*. Relative expression of *PGRP-LB* (**A**), *PGRP-SB*_1_ (**B**), and *PGRP-SC*_2_ (**C**) after dsRNA injection at different time points with whole body samples. All error bars represent the SEM of the mean of three independent biological replicates. Statistical analysis was based on Student’s *t*-test. * *p* < 0.05; ** *p* < 0.01. The relative gene expression data were analyzed using a 2^−ΔΔCT^ method and the data were normalized to reference gene *Rpl32*.

To evaluate the RNAi efficiency of *BdPGRPs*, we then monitored the expression of transcripts *BdPGRP-LB*, *BdPGRP-SB*_1_ and *BdPGRP-SC*_2_ in whole body samples at different days post-dsRNA injection (DPI). The results showed that the expression of *BdPGRP-LB* was significantly reduced, by 75.3%, 81.4% and 66.9% in the *ds-BdPGRP-LB* injection group at 1, 3 and 5 DPI when compared to the control *ds-egfp* group (Figure 5A); the expression of *BdPGRP-SB*_1_ was significantly reduced, by 87.4%, 88.2% and 93% in the *ds-BdPGRP-SB*_1_ dsRNA injection group at 1, 3 and 5 DPI (Figure 5B); and the expression of *BdPGRP-SC*_2_ was significantly reduced, by 33.7%, 28.5% and 39% in the *ds-BdPGRP-SC*_2_ dsRNA injection group at 1, 3 and 5 DPI (Figure 5C).

### 3.5. The Negative Regulatory Roles of BdPGRPs in Imd Pathway

**Figure 6:**
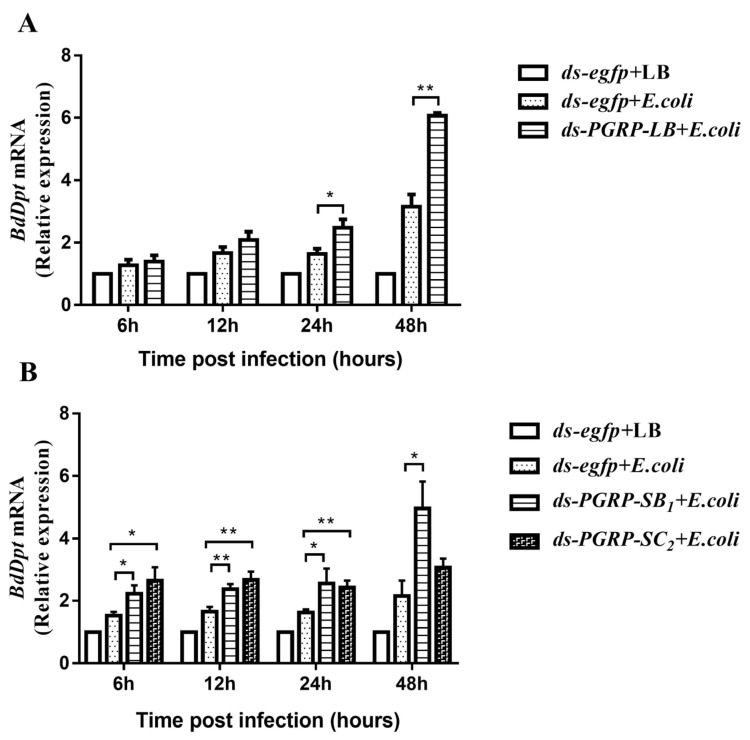
Antimicrobial peptide gene expression in *BdPGRPs* RNAi flies after bacterial challenges. (**A**,**B**) Injury infection with *E. coli* induced a higher *Diptericin* (*Dpt*) expression in *BdPGRPs* RNAi flies than in the *ds-egfp* dsRNA injection flies. The data are expressed as the mean ± SEM, and the mean refers to the average of at least three replicates for each sample. Statistical analysis was based on Student’s *t*-test. * *p* < 0.05; ** *p* < 0.01. The relative gene expression data were analyzed using a 2^−ΔΔCT^ method and the data were normalized to reference gene *Rpl32*.

To analyze the potential roles of *BdPGRP-LB*, *BdPGRP-SB*_1_ and *BdPGRP-SC*_2_ in the Imd pathway of *B. dorsalis*, we infected ds-*egfp*, *ds-BdPGRP-LB*, *ds-BdPGRP-SB*_1_ and *ds-BdPGRP-SC*_2_ treated flies with *E. coli* and measured *Dpt* transcript levels as a readout for Imd pathway activation. At 24 h and 48 h post *E. coli* infection, there was a 1.5 and 1.92-fold enhanced expression of *Dpt* in the infected *ds-BdPGRP-LB* group, respectively, compared to the infected *ds-egfp* group (Figure 6A). There was a 1.43–2.3-fold increase in expression of *Dpt* in the infected *ds-BdPGRP-SB*_1_ group compared to the infected *ds-egfp* group at 6, 12, 24 and 48 h post *E. coli* infection (Figure 6B). The knockdown of *BdPGRP-SC*_2_ led to a 1.74, 1.62 and 1.49-fold enhanced expression of *Dpt* at 6, 12 and 24 h post *E. coli* infection, respectively, as compared with infected *ds-egfp* group (Figure 6B). These results indicate that silencing of either *BdPGRP-LB*, *BdPGRP-SB* or *BdPGRP-SC*_2_ will induce overactivation of the Imd pathway upon bacterial infection, as all these three *BdPGRPs* perform negative regulatory roles in regulating *AMPs* gene expression in the Imd pathway of *B. dorsalis*.

### 3.6. BdPGRPs RNAi Decreased Flies Survival Rate after Bacterial Challenge

**Figure 7:**
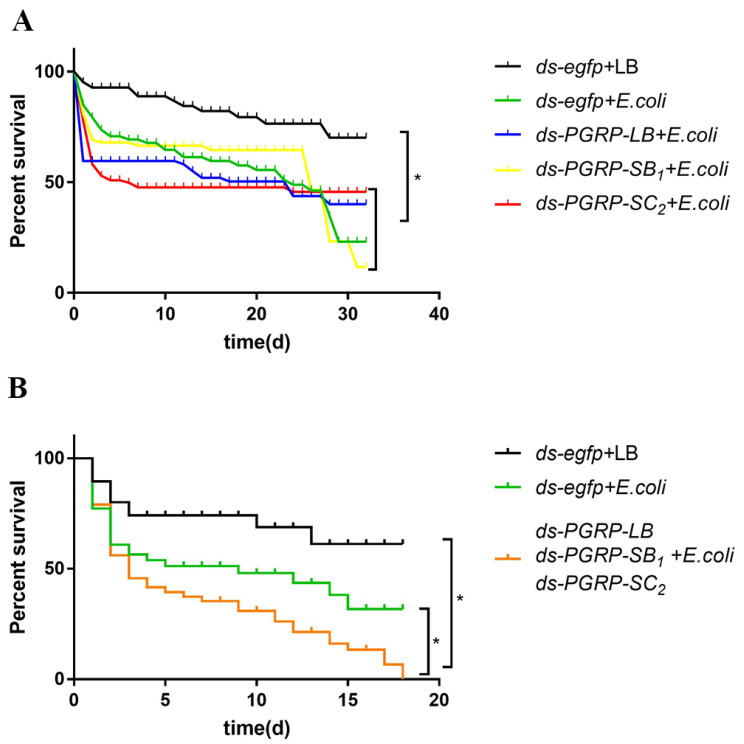
Survival rate of *B. dorsalis* after *BdPGRPs* RNAi followed by *E. coli* infection. (**A**) Three *BdPGRPs* were knocked down separately. (**B**) Three *BdPGRPs* were knocked down at the same time. Statistical analysis was based on Log-rank (Mantel–Cox) test (* *p* < 0.05).

After 24 h post dsRNA injection, flies that were alive in the control and treatment groups were individually challenged with *E.* *coli* by inoculation with bacteria in the thorax. From the results, we observed that the survival rate of infected flies was significantly lower than in the control group (Figure 7A). However, when *BdPGRP-LB, BdPGRP-SB*_1_ and *BdPGRP-SC*_2_ were silenced individually this did not affect survival status when compared with *ds-egfp* flies upon bacterial challenge (Figure 7A). Considering the functional redundancy of *BdPGRPs* in *B. dorsalis*, we knocked down of all three *BdPGRPs*. The results showed that the survival rate of the *ds-BdPGRPs* group was significantly reduced compared with the *ds-egfp* group after *E. coli* infection (Figure 7B). The median survival of *ds-egfp* group was nine days, while in the *ds-BdPGRPs* group it was shortened to three days.

## 4. Discussion

The PGRP family has been thoroughly studied in the last decade. PGRPs are evolutionally conserved proteins involved in the recognition and degradation of peptidoglycans, a cell wall component of bacteria [34]. PGRPs are involved in many immune processes ranging from initiation to termination of host immune activity; however, most research work has been concentrated in model animals such as in mice and *Drosophila* [12,23,35,36,37]. Here, we have characterized the immunological role of three PGRP family genes, *BdPGRP-LB*, *BdPGRP-SB*_1_ and *BdPGRP-SC*_2_, after inoculation with the Gram-negative bacterium *E. coli* in *B. dorsalis.* After applying RNAi methods to knock down *BdPGRPs* followed by Gram-negative bacterial infection, *Dpt*, a marker of IMD pathway activation, showed a significant increase compared with the *ds-egfp* group. The survival rate of the *ds-BdPGRPs* group was significantly reduced compared to the *ds-egfp* group after *E. coli* infection. Our results showed that *BdPGRP-LB*, *BdPGRP-SB*_1_ and *BdPGRP-SC*_2_ performed vital negative roles in regulating expression of *AMPs* in the Imd pathway of *B. dorsalis*, and maintaining the normal function of these three *BdPGRPs* is critical to host health when faced with bacterial challenge.

In this study, the results of the protein prediction indicated that *BdPGRP-LB, BdPGRP-SB*_1_ and *BdPGRP-SC*_2_ all have type 2 amidase domains, which suggests that *BdPGRP-LB, BdPGRP-SB*_1_ and *BdPGRP-SC*_2_ have amidase activity. PGRPs which have type 2 amidase domains have been confirmed to have important roles in innate immunity, not only in the model specie *Drosophila* [10] but in other insects such as *Tenebrio Molitor* [38], *Anopheles gambiae* [39], and *Nilaparvata lugens* [40]. This may indicate that the structure and function of PGRPs is highly conserved. All amidase-active PGRPs have a conserved Zn^2+^-binding site in the peptidoglycan-binding groove, which is also present in bacteriophage type 2 amidases and consists of two histidines, one tyrosine, and one cysteine [10]. The results on expression patterns showed that *BdPGRP-LB* and *BdPGRP-SC*_2_ were very highly expressed in the midgut of adults, while the *BdPGRP-SB*_1_ gene was mainly expressed in the fat body. The tissue expression profiling of *BdPGRPs* is similar with previous reports in other insects [8,10,26,41]. The fat body is the major tissue that generates AMPs to hemolymph in the systemic immune system, and intestinal epithelial cells produce AMPs to inhibit the overgrowth of pathogenic bacteria in gut lumen [6]. In the fat body of insects, characterized immune genes are induced by microbial infection and encode antimicrobial peptides which are then released into the hemolymph to defeat invading pathogens [42]. Updated research reveals that fat body tissues also synthesize and secrete some TEP and TOP peptides, which aid hemocyte phagocytosis [43]. Insects gut continually come in contact with microbiota, which generates a delicate intestinal immune response which must tolerate the presence of gut microbiota and dietary microorganisms while responding to and eliminating potential pathogens [13]. *BdPGRP-LB, BdPGRP-SB*_1_ and *BdPGRP-SC*_2_ all have highly conserved type 2 amidase domains and are highly expressed in immunocompetent tissues in *B. dorsalis,* indicating that *BdPGRPs* probably participate in the immune response of *B. dorsalis*, as in other insects.

In our study, injury infection with Gram-negative bacteria *E. coli* induced significantly higher transcript levels of *BdPGRP-LB* and *BdPGRP-SB*_1_ simultaneously. A similar immune expression of *PGRP-LB* has also been observed in *Drosophila*, where the expression of *PGRP-LB* was increased significantly following septic injury with *E. carotovora* [44]. *PGRP-SB*_1_ is strongly induced with injection of bacteria containing DAP-type PGN, which activates the Imd pathway [16]. Unexpectedly, there was a dramatic decrease in *BdPGRP-SC*_2_ expression upon systemic infection with *E. coli*. Similar results were observed in *Musca domestica* larvae; *MdPGRP-SC* cannot be induced when challenged by *E. coli* or *S. aureus* [26]. Based on the high expression of *PGRP-SC* in the guts of other insects [8,26], it is possible that *PGRP-SC* exerts its immune function in the gut. Stress stimulation can induce the transcription factor Foxo to help the host adapt to an adverse situation [45]. Guo et al. (2014) showed that chronic activation of the transcription factor Foxo reduces expression of *PGRP-SC*_2_ in *Drosophila* [15]. Therefore, it is plausible that the injury infection may induce the expression of *BdFoxo*, then decrease the expression of *BdPGRP-SC*_2_; however, further experiments are needed to reveal this phenomenon and its underlying mechanism. The knockdown of any *BdPGRP-LB*, *BdPGRP-SB*_1_ and *BdPGRP-SC*_2_ in flies will result in overactivation of the Imd signaling pathway upon bacterial challenge. The roles of *BdPGRP-LB* and *BdPGRP-SC*_2_ in *B. dorsalis* were consistent with previous findings in *D. melanogaster* that *PGRP-LB* and *PGRP-SC*_2_ act as important negative regulators of the Imd pathway [13,44,46,47]. After septic injury with the Gram-negative bacterium *Erwinia carotovora carotovora 15* (*Ecc15*), *PGRP-LB* deletion mutant flies had stronger and more sustained immune response than wild-type flies as measured by the expression of the antibacterial peptide gene *Diptericin* (*Dpt*), a readout of the Imd pathway. In contrast with the Zaidman-Rémy et al. (2011) report in *D. melanogaster* that injection of Gram-negative bacteria *Ecc15* did not affect the AMPs expression in *PGRP-SB* null mutant [16], the silencing of *BdPGRP-SB*_1_ in *B. dorsalis* induced enhanced expression of *Dpt* compared with the *ds-egfp* group after bacterial challenge. The discordance may be caused by different insect species having distinct catalytic PGRPs to regulate their systemic immune response. In *Drosophila*, three isoforms of PGRP-LB have two distinct functions; the PGRP-LB^PC^ isoform is required to control the systemic response in the fat body, while PGRP-LB^PA^ and PGRP-LB^PD^ isoforms show the immune function only in gut [48]. In our results, we found that each of the three *BdPGRPs* performed its individual negative function in the systemic Imd pathway of *B. dorsalis*, because the immune phenotype caused by the absence of one of the three *BdPGRPs* cannot be compensated by the other two. Collectively, our results showed that these three PGRP family genes act as negative regulators in the systemic immune response of *B. dorsalis* by dampening the activation of the Imd pathway.

A tight balance between initiation and resolution in the control of inflammation is very important in animals, as both the absence and overactivity of immune response are harmful to the host [46]. Our results showed that the survival rate of the *ds-BdPGRPs* group was significant reduced compared with the *ds-egfp* group. However, the death events were mostly observed shortly after infection and mid-to-late post-infection. Death in the short term may be caused by an overreaction of the immune system [13], while death in the middle and late stages may be caused by excessive energy consumption in response to infection [49]. Noncatalytic PGRPs are crucial for the sensing of bacteria in insects such as in *Drosophila*, and catalytic PGRPs play a vital role in hydrolyzing peptidoglycan by cleaving the amide bond [13]. The bacterial infection induced the expression of *PGRP-LB*, *PGRP-SB*_1_ and *PGRP-SC*_2_ to degrade PGN and repress the activation of *PGRP-LC*, which reportedly is the major receptor of the Imd pathway [22], in order to ensure that the immune response is at an appropriate level. This negative regulation integrates into the sensitive immune regulation mechanism of insects, which keeps pathogenic bacteria below the level where they can cause harm and ensures that the host will not be harmed by an overactive immune response.

## Figures and Tables

**Table 1:** Primers used in RT-PCR and qRT-PCR.

Primer	Sequence (from 5′ to 3′)	Purpose
*PGRP-SC2* 5′RACE outer	CCTTAGCGGCAGCAATCT	RACE
*PGRP-SC2* 5′RACE inner	CCACGACCCTCATACACT	RACE
*PGRP-SC2* 3′RACE outer	GCAAGTGTATGAGGGTCG	RACE
*PGRP-SC2* 3′RACE inner	TTACTGCTCCACCCAAAC	RACE
Q*PGRP-LB* F	GCGTGGCTGGAATGACATTG	qRT-PCR
Q*PGRP-LB* R	CGGTCATTGTATTTGGGCGC	qRT-PCR
Q*PGRP-SB* F	TGGCATTGTCTTCATCGGCA	qRT-PCR
Q*PGRP-SB* R	CAGATAACCCTTTTGCACCGC	qRT-PCR
Q*PGRP-SC*_2_ F	GGGTCGTGGTTGGAGTACAG	qRT-PCR
Q*PGRP-SC*_2_ R	GATCTGAGCGGCTGTTGGAA	qRT-PCR
Q*RpL32* F	CCCGTCATATGCTGCCAACT	qRT-PCR
Q*RpL32* R	GCGCGCTCAACAATTTCCTT	qRT-PCR
Q*Diptericin* F	GCATAGATTTGAGCCTTGACACAC	qRT-PCR
Q*Diptericin* R	GCCATATCGTCCGCCCAAAT	qRT-PCR
*PGRP-LB* T7F	GGATCCTAATACGACTCACTATAGGATGCCCAGCGCCTGTTAC	dsRNA synthesis
*PGRP-LB* T7R	GGATCCTAATACGACTCACTATAGGTGCGGCCACGTCGTAATC	dsRNA synthesis
*PGRP-SB* T7 F	GGATCCTAATACGACTCACTATAGGTGTTTTGCGCTCAGGATCCA	dsRNA synthesis
*PGRP-SB* T7R	GGATCCTAATACGACTCACTATAGGTGGCCCAGCAGTGTGTAATT	dsRNA synthesis
*PGRP-SC*_2_ T7 F	GGATCCTAATACGACTCACTATAGGGGCTTTCAAGACTTTCCTC	dsRNA synthesis
*PGRP-SC*_2_ T7R	GGATCCTAATACGACTCACTATAGGAACCACGACCCTCATACAC	dsRNA synthesis
*EGFP* T7L	GGATCCTAATACGACTCACTATAGGACGTAAACGGCCACAAGTTC	dsRNA synthesis
*EGFP* T7R	GGATCCTAATACGACTCACTATAGGAAGTCGTGCTGCTTAATGTG	dsRNA synthesis

Primers starting with Q were used for qRT-PCR; the underlined sections indicate T7 polymerase promoter.

## Data Availability

The data presented in this study are available within this artivle.

## Supplementary Materials

The following supporting information can be downloaded at: https://www.mdpi.com/article/10.3390/cells11010152/s1, Figure S1: Prediction of *BdPGRPs*’ functional domains; Figure S2: Phylogenetic tree of peptidoglycan recognition proteins of *B. dorsalis* and other insects; Figure S3: Expression levels of AMPs in Imd pathway after *E. coli* challenge; Table S1: Primers used in supplementary experiments.
