# Supplementary material for: The Negative Regulative Roles of BdPGRPs in the Imd Signaling Pathway of Bactrocera dorsalis
Source: Cells. 2022 Jan 4;11(1):152. doi: 10.3390/cells11010152 (PMC8750024; doi:10.3390/cells11010152)
Supplement: Supplementary file 1 [file cells-11-00152-s001.zip › cells-1499903-supplementary.pdf]

Table S1 **Primers used in supplementary experiments.**

Primer	Sequence (from 5' to3')	Purpose
Q*Cecropin* F	GGCAAGAAAATTGAGCGGGT	qRT-PCR
Q*Cecropin* R	CCTTCAATGTTGCTGCCACA	qRT-PCR
Q*AttacinA* F	GTGGCAACCTTAATTGGGCG	qRT-PCR
Q*AttacinA* R	AGATTGGAACTTGCGCCGTA	qRT-PCR
Q*AttacinB* F	ACACGCTTGGACTTGACAGG	qRT-PCR
Q*AttacinB* R	ATGAGTCAATCCCAAGCCGG	qRT-PCR
Q*AttacinC* F	GAGTTGGCCGGTAGAGCAAA	qRT-PCR
Q*AttacinC* R	GTAGTCGCGTTGTCCACTCA	qRT-PCR

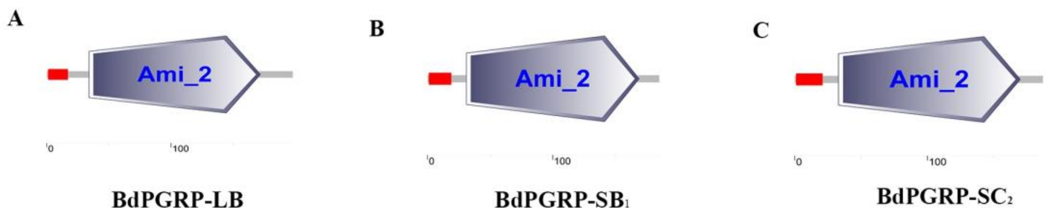

Figure S1. **Prediction of BdPGRPs' functional domains.** (A) PGRP-LB, (B) PGRP-SB1, (C) PGRP-SC2. Signal peptides are shown in red boxes. Ami_2 indicates amidase domain.

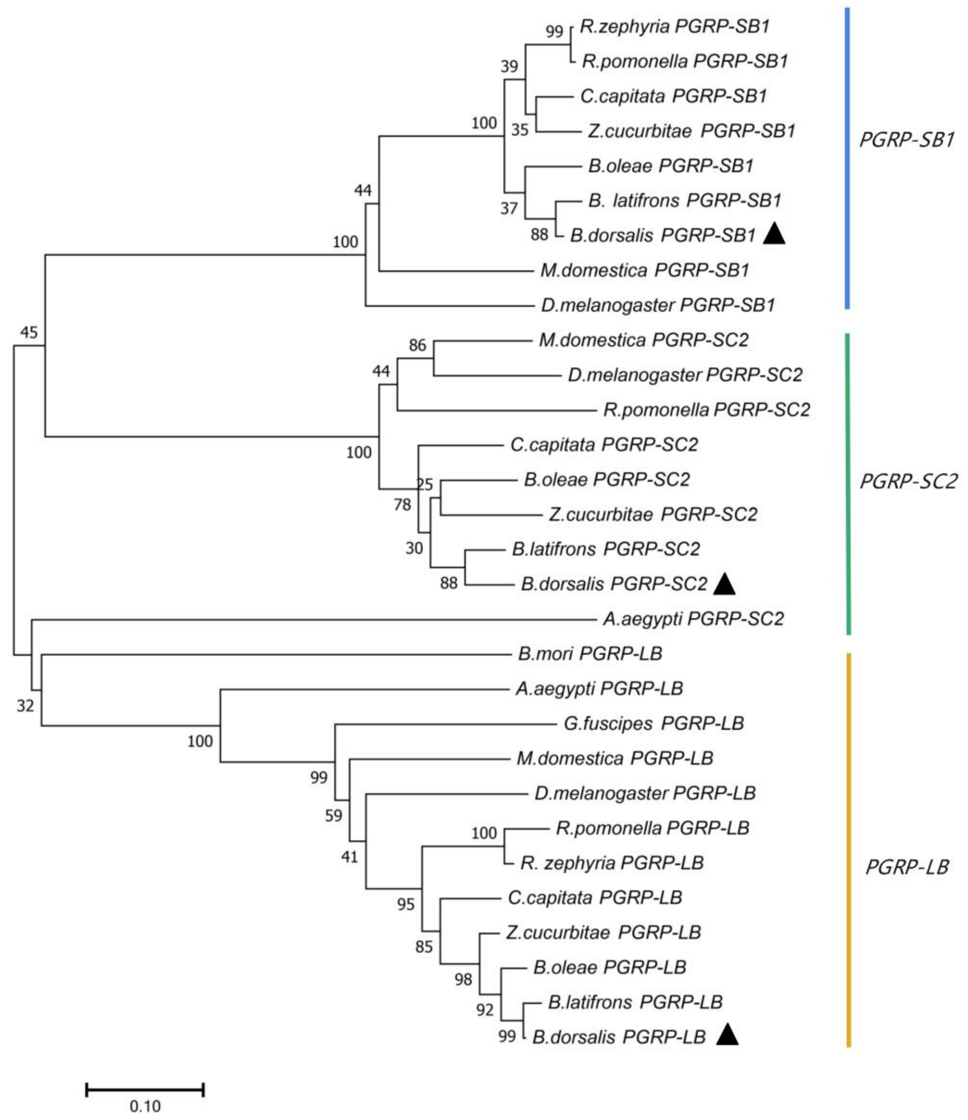

Figure S2. **Phylogenetic tree of peptidoglycan recognition proteins of *B. dorsalis* and other insects.** The genetic distance in phylogenetic neighbour-joining tree was calculated using the Kimura two parameter models. The result repeatability of NJ tree was validated through 1000 bootstraps. The numbers above the branches indicate the percentages of times that the species are grouped together in the bootstrap trees. The scale bar indicates the number of substitutions per site for a unit branch length. *BdPGRP-LB*, *BdPGRP-SB*$_1$, and *BdPGRP-SC*$_2$ were aligned with *Bactrocera latifrons PGRP-LB* (XP_018789449.1), *Bactrocera oleae PGRP-LB* (XP_014091181.2), *Zeugodacus cucurbitae PGRP-LB* (XP_011197144.1), *Ceratitis capitate PGRP-LB* (XP_004518089.1), *Rhagoletis zephyria PGRP-LB* (XP_017470705.1), *Rhagoletis pomonella PGRP-LB* (XP_036322481.1), *Aedes aegypti PGRP-LB* (XP_021709443.1), *Drosophila melanogaster PGRP-LB* (NP_731575.1), *Bombyx mori PGRP-LB* (XP_012548100.1), *Musca domestica*

PGRP-LB (XP_005180889.1), *Glossina fuscipes PGRP-LB* (ACI22620.1), *B. latifrons PGRP-SB* (XP_018789286.1), *B. oleae PGRP-SB* (XP_014099773.1), *Z. cucurbitae PGRP-SB* (XP_011181375.1), *C. capitate PGRP-SB* (XP_004537949.1), *R. zephyria PGRP-SB* (XP_017486043.1), *R. pomonella PGRP-SB* (XP_036336342.1), *D. melanogaster PGRP-SB* (CAD89135.1), *M. domestica PGRP-SB* (NP_001295929.1), *B. mori PGRP-SB* (XP_004929843.1), *B. latifrons PGRP-SC*$_2$ (XP_018798904.1), *B. oleae PGRP-SC*$_2$ (XP_014085196.2), *C. capitate PGRP-SC*$_2$ (XP_004520319.1), *Z. cucurbitae PGRP-SC*$_2$ (XP_011180165.1), *R. pomonella PGRP-SC*$_2$ (XP_036334551.1), *M. domestica PGRP-SC*$_2$ (XP_005184140.3), *D. melanogaster PGRP-SC*$_2$ (CAD89184.1), *A. aegypti PGRP-SC*$_2$ (XP_011492940.1), and *B. mori PGRP-SC*$_2$ (XP_004929814.1).

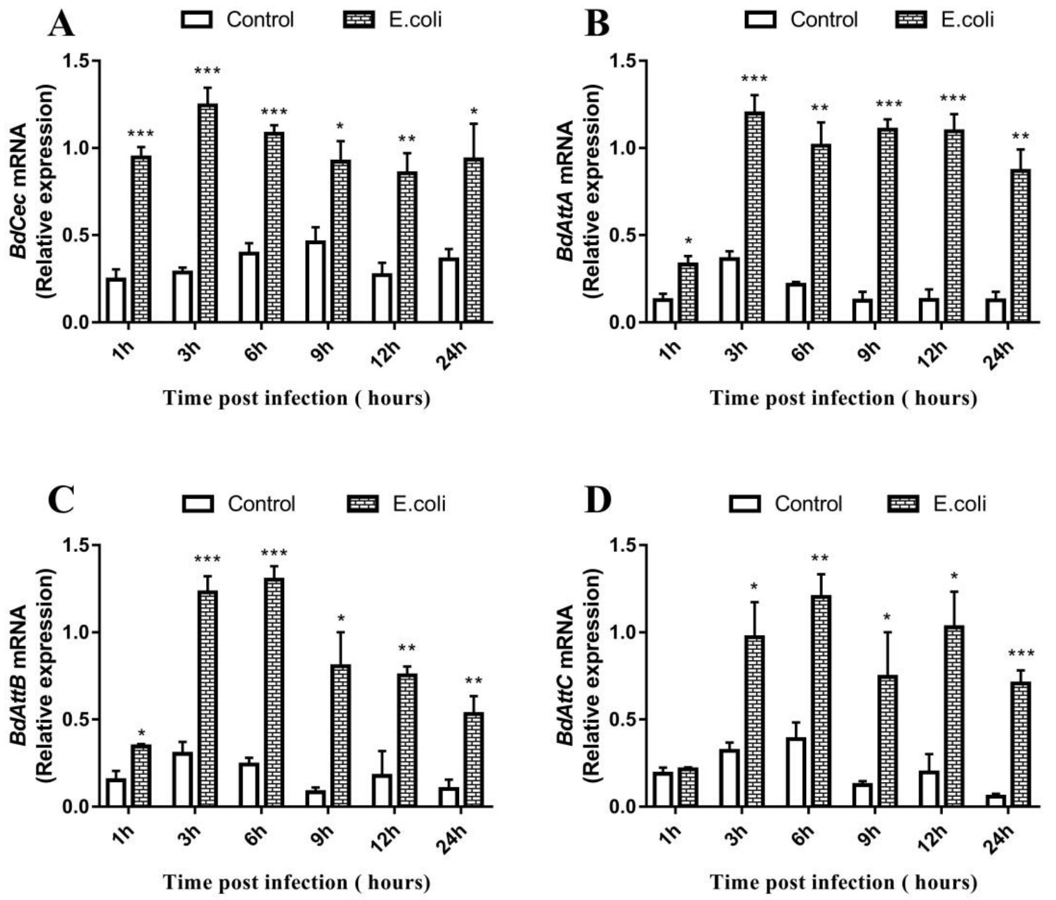

Figure S3. **Expression levels of *AMPs* in Imd pathway after *E. coli* challenge.** The expressions of (A) *Cecropin*, (B) *AttcinA*, (C) *AttcinB*, and (D) *AttcinC* in *B. dorsalis* were measured by qRT-PCR. The control was inoculated with LB medium. All error bars represent the SEM of the mean of three independent biological replicates. Statistical analysis was based on Student's t-test. *, P <0.05; **, P <0.01; ***, P <0.001; NS, no significant difference (P>0.05).
